# A Prospective Comparative Study of Pre-debridement and Post-debridement Cultures in Open Fractures of the Extremities

**DOI:** 10.7759/cureus.54778

**Published:** 2024-02-23

**Authors:** Jaga Dish, Arun H Shanthappa, Arvind Natarajan

**Affiliations:** 1 Department of Orthopaedics, Sri Devaraj Urs Medical College, Kolar, IND; 2 Department of Microbiology, Sri Devaraj Urs Medical College, Kolar, IND

**Keywords:** post-debridement, pre-debridement, surgical site infection, bacterial infection, open fracture

## Abstract

Introduction

Amputations and recurrent infections are two terrible outcomes of open fractures that can leave patients with permanent impairments. Rapid and effective treatment can protect patients from open fracture sequelae and the long-term financial burden these injuries frequently cause. Over 50% of open fractures are caused by high energy trauma, which most frequently happens in car accidents or severe falls. There hasn't been much research done on the first bacterial ecology of open fracture wounds in the Indian environment. Therefore, the need of the current assignment was to assess the effectiveness of pre-debridement and post-debridement culture in open fractures of the extremities.

Methodology

A prospective comparative study was carried out with 65 patients who were hospitalized from the OPD and Emergency departments at the R. L. Jalappa Hospital and Research Center. The time frame of study was between December 2020 and July 2022. Prior to the trial, each participant's written informed consent was obtained and strict protocol was followed in accordance with the Institutional Ethics Committee.

Results

Among the study participants, the majority of cases (26.15%) belonged to the 21-30 years of age group. A total of 14 participants belonged to the 41-50 years of age group. Out of the total, nine patients were aged less than 20 years. Out of the total, in pre-debridement culture the majority of cases had presence of growth of Staphylococcus aureus followed by Acinetobacter species, Enterobacter species and Pseudomonas species. Only six patients had growth of Klebsiella species. After debridement and treatment for bacterial infection, on subsequent culture examination, no growth was found among 61 patients. Although in four patients, there was presence of Pseudomonas species, Enterobacter species and Proteus microorganisms.

Conclusion

Although the validity of sequential cultures has been questioned in a number of investigations, this study has demonstrated that debridement cultures have a significant impact in postoperative infection. Debridement culture is therefore advised to offer information about the selection of antimicrobial medication, which when paired with a complete wound debridement will permit an early wound closure and better overall outcome functionally.

## Introduction

Amputations and recurrent infections are two terrible outcomes of open fractures that can leave patients with permanent impairments. Rapid and effective treatment can protect patients from open fracture sequelae and the long-term financial burden these injuries frequently cause. High-energy trauma is the most frequent cause of damage for open fractures, with over 50% occurring in auto accidents or falls from great heights [1,2]. High-energy open fractures are more common in young male patients than female patients, and these patients usually have concurrent injuries that are compounded by significant soft tissue damage [3]. If open fractures are not treated properly from the onset, they might result in substantial morbidity. Unfortunately, this is still the situation in several impoverished nations. In the past, these wounds would leave patients dealing with persistent infections, agony, and impairment, with many patients finally needing an amputation [3,4]. The care of patients with open fractures has significantly improved over time, mostly as a consequence of a greater understanding of the need for early therapy in addressing contamination and achieving early definitive closure and fixation.

Open fracture wounds are contaminated wounds, and the most common problem is the postoperative period which presents as infection [5]. The fracture pattern, the patient's co-morbidities, the presence of traumatized soft tissue, and the interval between the injury and therapy all affect the likelihood of bacterial infection. In their seminal 1976 paper, Gustilo and Anderson found that 70.3% of 158 open long-bone fracture wounds had a positive bacterial culture [6]. Eighty-three percent to 89% of fracture sites presented with first cultures were contaminated by bacteria, according to many investigators [7,8].

It has been quoted that determining the bacterial flora of the fracture site would enable logical and effective antibiotic treatment prior to starting antibiotic medication and making a final decision regarding wound management [9,10]. Because they are acquired in the community, contaminating bacteria in open fractures ought to be treatable by the majority of conventional antibiotics. In general, it is advisable to use an empiric antibiotic that has a broad spectrum activity against both Gram-positive and Gram-negative bacteria.

Delayed wound culture may reveal the original contaminating organism, which might indicate a technical debridement failure and enormous risk of postoperative infection. Reports contend that hospital-acquired microorganisms are to be blamed for surgical site infections [11]. This sparked a debate on the necessity and justification for acquiring first wound cultures, with some writers speculating that these cultures have little prognostic value for postoperative infection [12]. Others, however, assert that despite their lack of specificity, they have great sensitivity in detecting wounds that might get infected after surgery [13,14].

The first bacterial ecology of open fracture wounds in the Indian environment has not been extensively studied. Therefore, the need of the current assignment was to assess the effectiveness of pre-debridement and post-debridement culture in open fractures of the extremities.

## Materials and methods

A prospective comparative study was conducted among 65 patients with open fractures who came to the Department of Orthopaedics and Central Diagnostic Laboratory Services, Microbiology section of R. L. Jalappa Hospital (RLJH) attached to Sri Devaraj Urs Academy of Higher Education and Research Tamaka, Kolar, during December 2020 to July 2022. We received ethical approval from the Institutional Ethics Committee (IEC/633/2020-21/SDUMC/KLR).

Objectives of the study

Our objectives were 1) to assess the effectiveness of pre-debridement culture in identifying bacterial flora in open fractures of the extremities; 2) to evaluate the sensitivity of post-debridement culture in predicting postoperative infections in open fractures; and 3) to analyze the microbial profile and antibiogram patterns in open fractures in the Indian environment.

Sample size calculation

A study by Naique et al. [15] found a 9% difference in infection rates between pre- and post-debridement, assuming a 30% anticipated proportion in the population and an alpha error of 1% with 90% power.

Z^2^1−𝑎⁄2(1−𝑝)/d^2^ equals n, where p: 5.4% infection rate expected proportions and d: 8% absolute precision. 1.96 (95% confidence level) is the intended confidence level, denoted by 1-α/2; Z: interval of confidence.

With a 20% dropout rate anticipated during the trial, an estimated 54 people will make up the sample. The sample size in the end was 54 + 11 = 65.

The study included all patients who, within six hours of trauma, presented with open fractures of the upper and lower extremities (Gustilo-Anderson classification I, II, IIIA, IIIB). Individuals who have already had surgery or wound debridement performed before coming to the hospital or already underwent an antiseptic wound dressing and also, already received antibiotics were excluded.

Method of data collection

Patients whose profile fitted that of the inclusion criteria were considered in our study. The patient and their attendants provided the working proforma with the following information: demographic information, the date and time of the accident, the mechanism of the injury, time since the patient's injuries and admission to RLJH.

The injured limb(s) were splinted, the wound was examined for size and extent, both soft tissue and bone health were checked, and the amount of contamination was noted for all patients who had trauma assessment and appropriate management in the emergency room. Gustilo Anderson's classification of open fractures served as the basis for a tentative classification of all wounds. All the patients were given tetanus toxoid and the limb was splinted. The wound was covered by a sterile saline-soaked gauze. Under complete aseptic precautions the wound was first cleaned with sterile normal saline and then a wound swab was taken and sent to Central Diagnostic Laboratory Services, Microbiology section. This was considered to be the pre-debridement sample. Then a thorough wound toileting is carried out in the operation theatre using 6-10 liters of saline under complete asepsis, following which another swab was taken deep within the wound. This was considered to be the post-debridement sample which was also sent to Central Diagnostic Laboratory Services, Microbiology section. The wound was then debrided under anesthesia and the open fracture was classified depending on findings. The bony injury is stabilized depending on criteria such as soft tissue coverage, comminution, contamination, and periosteal stripping. The soft tissue wounds were dressed as necessary. Further management of the fracture was carried out based on standard protocol/guidelines.

Swabs were immediately streaked onto blood agar and MacConkey agar medium upon receipt (both pre- and post-debridement), incubated aerobically at 37°C for 24-48 hours, and checked for any bacterial growth. Standard bacteriological techniques were used to further identify any bacterial growth, and relevant biochemical assays were run in accordance with the standard protocol (like Gram stain, catalase, coagulase, oxidase, indole, citrate, urease, mannitol motility and triple sugar iron tests) [16,17].

All patients were started on antibiotics after pre-debridement culture samples based on hospital protocol. A prophylactic antibiotic (cefuroxime) was given for three days, which is based on our hospital antibiotic policy and later escalate/de-escalate antibiotics based on the culture sensitivity report of pre and post-debridement cultures. If no growth occurs antibiotics are stopped on the fifth day.

The patient during hospital stay was assessed clinically for signs of infection and repeat cultures were sent if infection was found to be present. Clinical indicators taken into account that suggested a wound infection were localized temperature increase, pain, sero-sanguinous discharge, abscess collection, frank pus, foul odor, necrosis of the graft or flap, and fever with chills.

The patient's wound was inspected regularly and dressing was done using aseptic measures. If there was any evidence of infection the wound sample was again sent for culture and antibiotics started and secondary definitive soft tissue and bony procedure was done to obtain coverage. Once soft tissue coverage was established and the patient had no evidence of infection, the patient was discharged from hospital. The microbial profile and antibiogram pattern were documented and analyzed.

Written informed consent was taken prior to the study of each participant. All ethics morals were followed in the study. The composed data was utilized only for the anticipated purpose of the study. The dignity and welfare of participants were shielded at all times from an ethical point of view. The authors got the study participants' permission to use their true identities in the report analysis, and the research data was kept suppressed throughout the study.

Data entry and analysis

Open fractures were classified based on Gustilo-Anderson classification (Table 1). Data was collected from case proforma form and entered into Excel 2016 (Microsoft, Redmond, WA, USA). Data analysis was done in SPSS Software version 26 (IBM Corp., Armonk, NY, USA).

**Table 1:** Gustilo-Anderson classification for open fractures

TYPE	DESCRIPTION
Type I	Clean wound <1cm in diameter with simple fracture pattern and no skin crushing.
Type II	A laceration >1cm and <10cm without significant soft tissue crushing. The wound bed may appear moderately contaminated.
Type III	An open segmental fracture or a single fracture with extensive soft tissue injury >10cm. Type III injuries are subdivided into three types
Type III A	Adequate soft tissue coverage of the fracture despite high energy trauma or extensive laceration or skin flaps.
Type III B	Inadequate soft tissue coverage with periosteal stripping.
Type III C	Any open fracture that is associated with vascular injury that requires repair.

## Results

Among the study participants, the majority of cases (26.15%) belonged to the 21-30 years of age group. A total of 14 participants belonged to the 41-50 years of age group. Out of the total, nine patients were aged less than 20 years. Only two patients were aged more than 80 years. Participants in the study had an average age of 40.11+17.5 years as mentioned in Table 2.

**Table 2:** Age-wise distribution among study participants (n=65)

Age group (in years)	Frequency (%)
<20	9 (13.84)
21-30	17 (26.15)
31-40	10 (15.3)
41-50	14 (21.5)
51-60	7 (10.7)
61-70	4 (6.15)
71-80	2 (3)
>80	2 (3)

**Figure 1:**
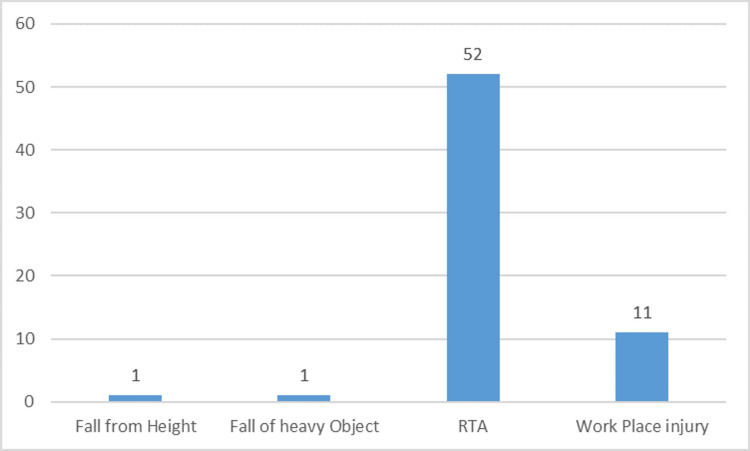
Mode of injury among study participants (n=65) RTA: road traffic accident

Out of the total, in one patient mechanism of injury was fall from height, in 80% of participants mode of injury was road traffic accident and 11 patients were injured at work place. The remaining one patient was injured due to fall of a heavy object (Figure 1).

Out of the total, in pre-debridement culture, the majority of cases had presence of growth of Staphylococcus aureus (15.3%) followed by Acinetobacter, Enterobacter, and Pseudomonas. Only six patients had growth of Klebsiella as mentioned in Table 3.

**Table 3:** Pre-debridement growth of microorganisms among study participants (n=65)

Pre-debridement growth	Frequency (%)
Acinetobacter species	9 (13.8)
Enterobacter species	9 (13.8)
Staphylococcus aureus	10 (15.3)
Klebsiella species	6 (9.2)
Pseudomonas species	9 (13.8)
None	22 (33.8)

After post-debridement, only 13 patients' cultures found the growth of microorganisms. The most common species found was Pseudomonas followed by Acinetobacter, Proteus vulgaris, Klebsiella and Enterobacter as mentioned in Table 4.

**Table 4:** Post-debridement growth of microorganisms (n=65)

Post-debridement growth	Frequency (%)
Acinetobacter species	2 (3)
Enterobacter species	4 (6.2)
Proteus vulgaris	2 (3)
Klebsiella species	2 (3)
Pseudomonas species	3 (4.6)
None	52 (80)

**Figure 2:**
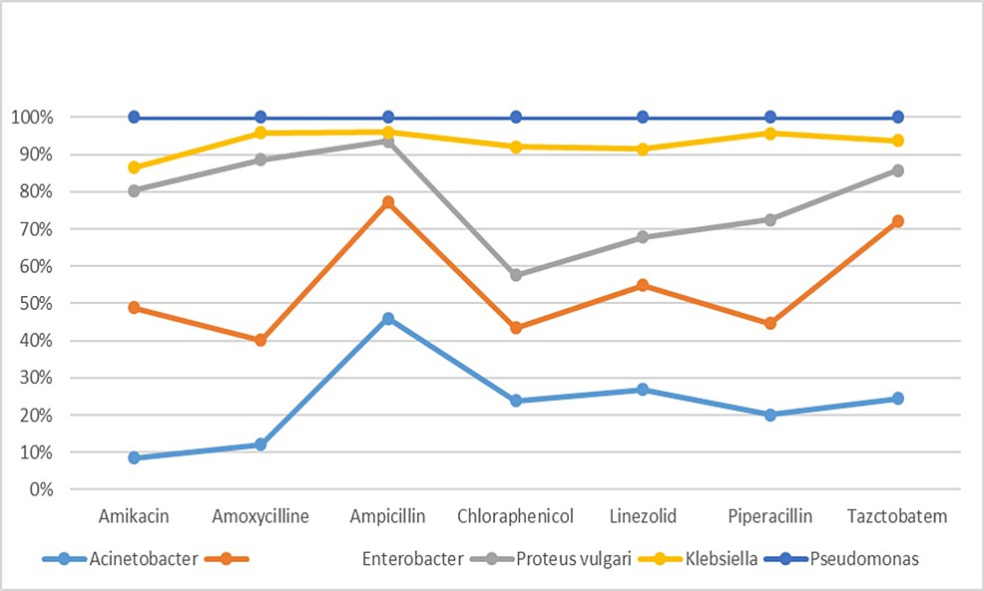
Antibiotic sensitivity among study participants (n=65)

Overall the sensitivity was higher to piperacillin and tazobactam followed by less sensitivity to amikacin as mentioned in Figure 2.

Out of the total study population, 43 (66.15%) cases had positive cultures in pre-debridement samples and 13 (20%) cases had positive cultures in post-debridement samples. Higher infective rate is positive in type IIIA and IIIB where out of 10 (15.4%) cases nine (13.84%) cases were positive and four (6.2%) cases out of four (6.2%) respectively as mentioned in Table 5.

**Table 5:** Distribution of open fracture on basis of Gustilo Anderson classification and their relation with pre- and post-debridement infection

Gustilo Anderson Classification	Frequency of injured cases (%)	Frequency of pre-debridement infected cases (%)	Frequency of post-debridement infected cases (%)
I	22 (33.84)	12 (18.46)	-
II	28 (43.1)	18 (27.69)	-
IIIA	10 (15.4)	9 (13.84)	9 (13.84)
IIIB	4 (6.2)	4 (6.15)	4 (6.15)
IIIC	1 (1.5)	-	-

## Discussion

Open fractures can be modest to severe in soft tissue and skeletal injury, both of which reduce the vascularity of the surrounding tissue. They are frequently the result of extreme-velocity injuries [18]. Due to the transmission of the bacteria, all open fracture wounds should be considered infected fracture sites and the environment beyond [19]. Exogenous or endogenous wound contamination can happen in traumatic wounds. Depending on whether contamination occurs at the moment of injury, immediately after damage, or 24 hours or more after injury, it may be categorized as primary or secondary [19,20].

Cultures obtained from various fracture locations have yielded recovered organisms belonging to numerous taxa. Nonetheless, it appears possible that the environment, feces, or skin may have contaminated the fracture sites due to the recurring isolation of specific aerobic or anaerobic microbial isolates. Numerous publications have suggested that many infections in open fractures are nosocomial, based on the sorts of organisms causing infections compared to those found on initial wound cultures [20]. The results of the test should enable the doctor to modify the course of therapy in some manner to enhance the outcome, or at the very least, to predict the severity or future course of the patient's illness. Due to the variance in bacterial frequency in various countries, as well as between hospitals within the same nation, wound-infecting pathogens vary [21].

In the present study, the majority of cases (26.15%) belonged to the 21-30 years of age group. A total of 14 participants belonged to the 41-50 years of age group. Out of the total, nine patients were aged less than 20 years. Only two patients were aged more than 80 years. Participants in the study had an average age of 40.11+17.5 years. In the research of Khatod et al. [20], the mean age of study participants was 29.7+15.4 years as mentioned in Table 6.

**Table 6:** Comparison of mean age of patients among various studies

	Mean age (in years)
Present study	40.11
Khatod M et al. [20]	29.7

In the present study, 15% of the participants were women and 85% were men. Compared to women, there were more men. Studies by Singh et al. [21] and Fernandes et al. [22] found similar results.

One patient out of the total had a fall from a height as their cause of injury, road traffic accidents caused 80% of patient injuries, and 11 patients had workplace injuries. The remaining patient was injured due to a fall of a heavy object.

Roth et al. [23] indicated that assault (3.3%), falls from height (6.7%), firearm injuries (15%), and roadside accidents (68%) were the most common modes of injury as mentioned in Table 7.

**Table 7:** Comparison of mode of injury among various studies Percentage(%) among various studies

Mode of Injury	Present study	Roth et al. [23]
Fall from height	3.1%	6.7%
Road traffic accident	80%	68%
Injured at workplace	16.9%	18.3%

In the present study, in pre-debridement culture majority of cases had presence of growth of Staphylococcus followed by Acinetobacter, Enterobacter, and Pseudomonas. Only six patients had growth of Klebsiella. After post-debridement, there were only 13 patients whose culture found the growth of microorganisms. The most common species found was Pseudomonas followed by Acinetobacter, Proteus vulgaris, Klebsiella, and Enterobacter. After debridement treatment for bacterial infection, on subsequent culture examination, no growth was found among 61 patients. However, in four patients, there was presence of Pseudomonas, Enterobacter and Proteus microorganisms.

E. coli made up the majority of the Gram-negative bacteria in the study by Lakshminarayan et al. [24], with Pseudomonas (13.6%), Proteus (4.6%), and Klebsiella (10.2%) of the population growing next; S. aureus and hemolytic streptococci, on the other hand, were isolated from 40.9% and 2.3% of the population, respectively.

Johnson et al. [25] detected S. aureus in 18.2% of the Gram-positive population. Acinetobacter (19.7%) was the most abundant Gram-negative bacterium, followed by Pseudomonas (12.1%). In comparison to 4.54% of samples that had Klebsiella and E. coli, 9.09% of samples had Enterobacter growth.

According to Agrawal et al. [26] the two most common Gram-negative bacteria were E. coli (34.2%) and Pseudomonas (26.1%). Klebsiella growth was found in 8% of cases, followed by Proteus in 6.3% as mentioned in Table 8.

**Table 8:** Comparison of pre-debridemental growth of various microorganisms Percentage(%) among various studies

Pre-debridement growth	Present study	Lakshminarayan et al. [24]	Johnson et al. [25]	Agrawal et al. [26]
Acinetobacter species	13.8%	-	19.7%	-
Enterobacter species	13.8%	-	9.09%	34.2%
Staphylococcus aureus	15.3%	40.9%	18.2%	-
Klebsiella species	9.2%	10.2%	4.54%	8%
Pseudomonas species	13.8%	13.6%	12.1%	26.1%
Proteus	-	4.6%	-	6.3%
None	33.8%	-	-	-

Limitations

We think a multicenter randomized trial would be essential to more thoroughly evaluate the relationship between the occurrence of infection and time, but ethical constraints prevent this kind of analysis. Other factors that affect the likelihood of infection include those related to the patient (smoking, diabetes, and other comorbidities), the type of fracture (the severity and location of the lesion), and the type of surgery (the surgeon's experience, the aggressiveness with which devitalized tissues are removed, and the type of synthesis recommended).

Future direction for study

This study acts as a guidance for budding clinicians to make decisions and adhere to the initiation of prophylactic antibiotics followed by thorough wound debridement which helps in overall reduction of incidence of postoperative infection. 

## Conclusions

There is a considerable danger of infection and other problems from open fracture wounds. The treatment of open fractures focuses on timely wound closure, proper antibiotic medication, and efficient wound debridement. The management of infection depends heavily on diagnostic microbiology. When compared to pre-debridement cultures, cultures collected during debridement are found to be more sensitive in predicting the infection rate. Although the validity of sequential cultures has been questioned in a number of investigations, this study has demonstrated that debridement cultures have a significant impact in the prediction of postoperative infection. Debridement culture is therefore advised to offer information about the selection of antimicrobial medication, which when paired with a complete wound debridement will permit an early wound closure and better functional outcome.

## Appendices

**Table 9:** Proforma (Demographic details, culture reports)

Name	
Age/Sex	
Address	
Date and Time of injury	
Nature of injury	2 wheeler 3 wheeler 4 wheeler
Pedestrians	Peds + 2 wheeler Peds + 3 wheeler Peds + 4 wheeler
Fall height	
Assault	
Date and time of admission	
Nature of first aid	<6hrs/>6hrs
Tetanus prophylaxis	
Gas gangrene prophylaxis	
Splinting of limb	
Gustilo Anderson type	I II IIIA IIIB IIIC
Time of first aid to primary treatment	
Type of treatment	SOFT TISSUE Debridement SSG Flap Amputation BONE External fixation Internal fixation Plate and screws Nail
Special procedure	Vascular repair Nerve repair
OT duration	
Pre-debridement culture	
Sensitivity	
Post debridement culture	
Sensitivity	
Subsequent cultures	
Sensitivity	
Any comorbidities	
